# Design and implementation of the 6-C model of community engagement

**DOI:** 10.3389/fpubh.2025.1648108

**Published:** 2026-01-21

**Authors:** Karen Therese D'Alonzo, Shailja Mathur, Maya E. Joseph, Anju Wadhawan, Pam Hargwood, Teresa Clarita Vivar

**Affiliations:** 1 Rutgers School of Nursing, Newark, NJ, United States; 2 Department of Family and Community Health Sciences (FCHS), Rutgers Cooperative Extension of Somerset County, Newark, NJ, United States; 3 William Paterson University School of Nursing, Newark, NJ, United States; 4 Rutgers The State University of New Jersey School of Nursing, Newark, NJ, United States; 5 RWJ Library of the Health Sciences Rutgers, The State University of New Jersey, Newark, NJ, United States; 6 Lazos America Unida, New Brunswick, NJ, United States

**Keywords:** community engaged research (CER), community academic partnerships (CAPs), community engagement, stakeholder engagement, community-based participatory research (CBPR)

## Abstract

The term “community engaged research” (CER) has become popularized to describe attempts to promote community involvement in all phases of the research process. The aim of CER is that the people most directly impacted by the research topic become active participants in the research process. Historically, the term CER referred to scientific researchers and community members who work together to develop community academic partnerships (CAPs), improve research, and create better outcomes in communities, CER is most often conducted in the context of health issues, but need not be limited to that field. There are various forms of community engagement; many of them have demonstrated the potential to contribute to fostering health equity in a community. As more individuals and groups are invited to participate in CAPs and have their voices heard, the members embrace bi-directional communication and trust and share power. At the righthand side of the scale is community-based participatory research (CBPR), a more egalitarian form of CER, characterized by strong collaboration across the research spectrum. Although CBPR has become the most frequently cited example of CER, it can be difficult to build and sustain. Time commitments can be off-putting, both for researchers and community members. Research projects may be difficult to maintain without a steady stream of external funding. More recently, political factors may contribute to an erosion of trust in the CAP. There is an acknowledgment of multiple points of community engagement and the potential for important health contributions with these various forms of CER.

## Introduction

The phrase “community engaged research” (CER) has become popularized over the past two decades to describe attempts to promote community involvement in all phases of the research process. The aim of CER is that the people most directly impacted by the research topic become active participants in the research process (1). CER emerged from several fields, including social work, sociology, and public policy, with roots in social justice and civil rights movements. Historically, the term CER referred to scientific researchers and community members who work together to develop community academic partnerships (CAPs), improve research, and create better outcomes in communities, particularly for marginalized populations (2). Recently, the term “community engagement” has been expanded by some to signify “stakeholder engagement”, where stakeholders include not only community members, but also health care providers and health care systems; research participants; and patients (3). CER is most often conducted in the context of health issues (4), but need not be limited to that field. Related disciplines that utilize CER include education (5), citizen science (6), as well as environmental (7), and social sciences (8). CER can be used successfully in these fields, particularly when a transdisciplinary participative approach to problem solving is favored for problem definition and intervention planning.

**Figure 1:**
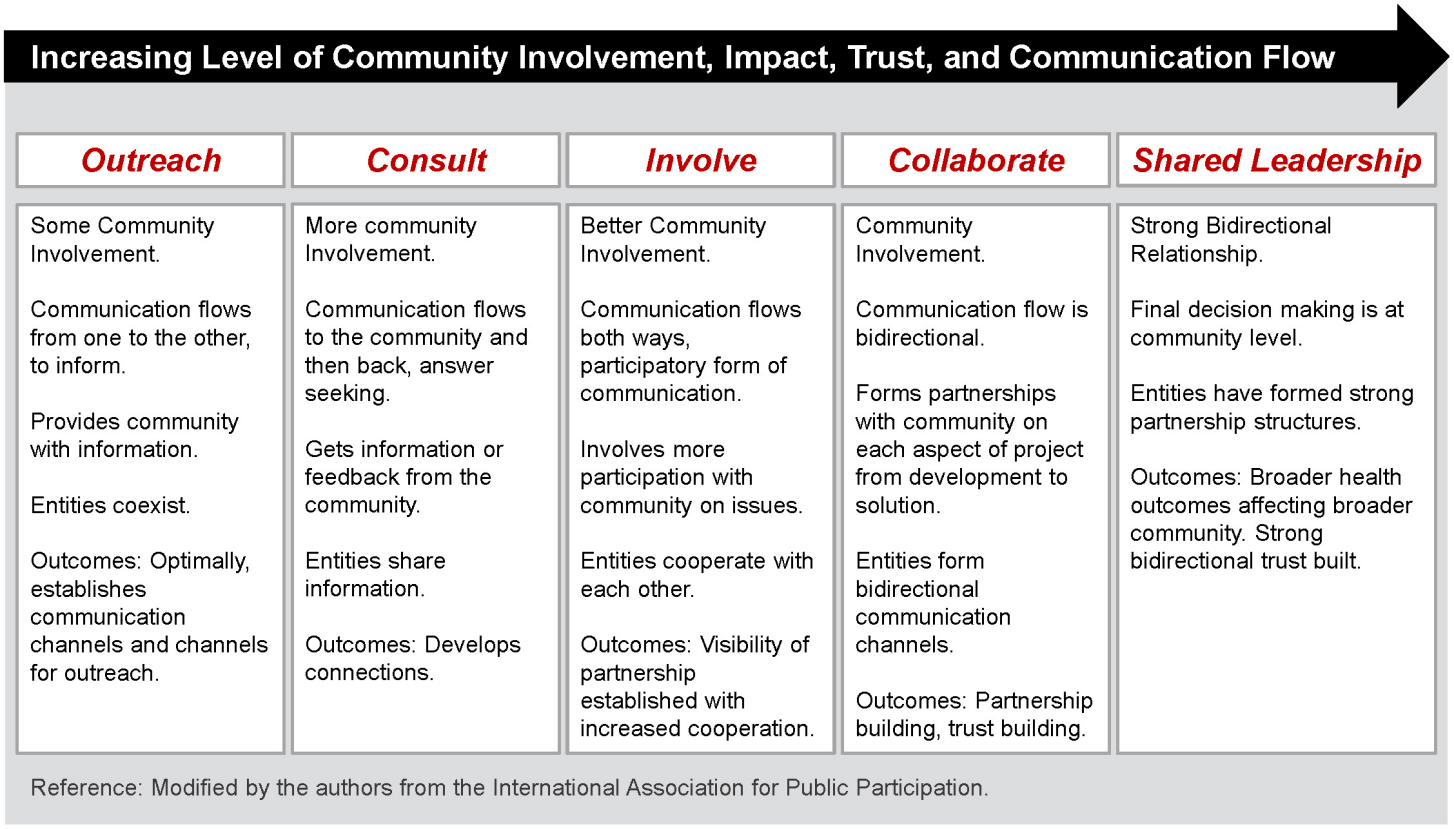
Continuum of community engagement. Community engagement continuum, developed by the clinical and translation science awards consortium (2011).

CER can be characterized as both a science and an art, where the art is used to apply and adapt the science in ways that fit the community and the specific engagement efforts (9). There are various forms of community engagement; (10–13) many of them have demonstrated the potential to contribute to fostering health equity in a community (14). CER is often conceptualized as a continuum, as seen in Figure 1, with increasing levels of community involvement, impact, trust, and communication to the right in the scale. As more individuals and groups are invited to participate in CAPs and have their voices heard, the members learn to embrace bi-directional communication and trust and share power. At the right hand side of the scale is community-based participatory research (CBPR), a more egalitarian form of CER, characterized by strong collaboration across the research spectrum (15). Although CBPR has become the most frequently cited example of CER (16), it can be difficult to build and sustain. Time commitments can be off-putting, both for researchers and community members. Research projects may be difficult to maintain without a steady stream of external funding. More recently, political factors may contribute to an erosion of trust in the CAP. Although CBPR, with its emphasis on a shared leadership style, is arguably acknowledged as the gold standard of CER (17), there is also an acknowledgment of multiple points of community engagement and the potential for important health contributions with these various forms of CER (1).

### Need for a new conceptual model

In addition to the impact of external factors on CAPs, the partnerships themselves may fail to address the cultural and social barriers that limit community participation. Existing community engagement models such as Arnstein's Ladder of Citizen Participation; (18) CBPR Conceptual Model (University of New Mexico); (19) International Association for Public Participation model (20) and the Healthy Flint Research Coordinating Center (HFRCC) CEnR Model (17) may not provide sufficient guidance to cultivate a sense of egalitarianism in the CAP. Three of these models are based on CER as a continuum (18, 20, 21), while the CBPR Conceptual Model addresses contents, partnership processes, intervention, and research. However, all of the these models utilize a “top down” approach and none specifically speak to: (1) the influence of culture in the formation of the CAP; (2) the community's sense of the quality of the CAP relationship or what could be done to improve it; and (3) how the community negotiates imbalances of power within the CAP. Recent modifications of the CBPR Conceptual Model and the HFRCC Model do allow for some input by community members but community members do not rate the quantity or the quality of key CAP behaviors.

To address these concerns, we created the “6C model of community engagement” based on 20+ years of collaborative work to address health equity challenges in immigrant communities. The model specifically examines six characteristics of community engagement: community health focus, collaboration, co-learning, cultural competence, critical consciousness, and capacity building. We will first describe our “impact stories” (21), our experiences creating and testing the 6C model, then explain how community and academic partners can use this model to strengthen CAPs and encourage progress toward a more shared leadership style.

### Impact stories

Impact stories are narratives that demonstrate how an organization (e.g., a CAP) can create positive change for individuals or communities. Impact stories can reflect the mutual experiences of community members and academic partners; this process of shared understanding and mutuality are key components in relational-cultural theory (RCT) (22). RCT is frequently aligned with social justice movements and is therefore an ideal framework for CER.

The principal author was introduced to CER methods while working with immigrant Latinas and their families in the early 2000s (23). She entered into a CAP with the Salvation Army to provide screening and health education activities to the Costa-Rican immigrant community. It was here that the imbalance of power inherent in many CAPs first became apparent. Faculty members benefit from CER through university-sanctioned mechanisms such as grant support, manuscripts, and scholarly presentations which can lead to promotion and tenure. Although there are examples of CER where community partners serve as consultants to the academic community (24), this is the exception rather than the norm. Community members rarely receive comparable accolades for participation in CER, which in turn can influence their participation in the CAP, especially in a low-income immigrant community. This imbalance of power is referred to as positionality; (25) the term describes the privileges conferred upon researchers as trained professionals and how knowledge is traditionally reproduced to confer privilege. Positionality acknowledges that a researcher's background can impact the full spectrum of their work, including partnering with community organizations. Keeping this in mind, the CAP decided that the next collaborative project would be a Photovoice study (26). Photovoice is a visual qualitative research method where individuals chronicle their lives and communities through photography and narration, creating a powerful story that can be used for advocacy and social change. Eight immigrant Hispanic women were given digital cameras and asked to photograph typical daily routines, including household activities, family/childcare and occupational responsibilities. Subjects then met to discuss their impressions. The women identified that working long hours outside the home with very little sleep was the major obstacle to regular exercise. Ironically, the women were working to contribute to their children's college education, yet neglecting their own physical and emotional needs, which is consistent with the Latinx concept of *marianismo*. Photovoice was used in this study as an innovative method to draw attention to the impact of cultural beliefs on health and social justice issues.

In a second study, the principal author partnered with *Lazos America Unida*, a 501(c)(3) non-profit organization in Central New Jersey, that advocates on behalf of the Latinx immigrant community. *Lazos* had previous experience working in a CAP at Rutgers and readily embraced the principles of collaboration and co-learning. Together, the principal author and *Lazos* co-designed and implemented a highly successful physical activity intervention for immigrant Latinas. In this study, the physical activity classes were facilitated and managed by *promotoras* (27). In this study, there were significant improvements in aerobic fitness, muscle strength and flexibility, and daily physical activity (PA) levels (*p* < 0.001). The only true dropouts after group assignment consisted of women who became pregnant during the study. This is impressive, as dropout rates in physical activity intervention have been long been reported to be as high as 50% (28). Overall, the study provides evidence that laywomen trained as *promotoras* can successfully deliver an intervention to increase PA among immigrant Latinas and become partners in community-level research. After the study finished in 2010, the *promotoras* were able to incorporate the study design into a self-sustaining community exercise program for women that ran for 14 years. Following a brief pause during the COVID-19 pandemic, this community program is up and running once again. The following year, the CAP took time to evaluate their partnership using the framework developed by the Clinical and Translational Science Award Consortium (29), and determined they were at the “Collaborate” level (Figure 1). Engaged in capacity building, two of the *promotoras* returned to community college to study nursing, while others elected to serve as co-authors on study-related manuscripts. In turn, the CAP taught other faculty members interested in CER best practices for managing these relationships.

*Lazos* continued to work with the principal author on a number of research projects involving cardiovascular risk reduction among Mexican immigrant women and their families. The work of the *promotoras* became more publicized and the principal author was approached to start a similar program in the Asian Indian (AI) immigrant community. Similar to previous CAPs, the research team began by talking to community members at a Hindu temple about their health concerns. Hypertension was noted as a topic of interest, so blood pressure screenings and health education sessions were initiated. Aided by the intervention of a long-term member of the AI immigrant community, a CAP was formed between the University and the temple and plans for a pilot study of cardiovascular risk factors was undertaken. Although the CAP was relatively new, it quickly developed into the “Involve” level. Eager to share power with the investigators, participants offered practical suggestions to improve the study. Cultural competence was fostered as study activities were planned around the Hindu holidays and holy days and a second site involving a Syro-Malabar Catholic church was added. AI community health workers/peer leaders were recruited to conduct Group Concept Mapping sessions (taught by the *promotoras*), which informed the format of the pilot cardioprotective intervention. A major topic of discussion at the brainstorming sessions was the need for stress management, which included a discussion of acculturative stress. The AI peer leaders helped those investigators who were not AIs to see how discrimination and oppression negatively impacted the lives of these immigrants and prevented them from engaging in health-promoting behaviors. This led the research team to include stress management strategies as an element of the cardioprotective intervention, suggesting this CAP was approaching the “Collaborate” level.

Based upon their experiences working in two immigrant communities, the authors determined that none of the existing community engagement models adequately addressed the role of culture in forming a CAP. For example, we found that some Latino immigrants were reluctant to form a partnership with the academic partner, which is a state university. They perceived the faculty members to be government employees, who might be required to identify and report undocumented individuals in the community. This misunderstanding was initially a major source of mistrust among community members. Another example arose in the AI community, following a discussion in a Hindu temple about a reluctance among participants to participate in CVD prevention activities. The AI community consultant reminded the research team that Hindus are “a people who believe they will be back,” suggesting that through reincarnation, they will have another chance to live healthy in their next life. This cultural perspective puts a very different spin on the idea of health promotion.

## Materials and methods

It was at this point that authors reflected on their 20+ years of collaborative work and the shortcomings of existing community engagement models (17–20). Three limitations were noted in particular:

- Few frameworks address the influence of culture in the formation and function of the CAP and how that impacts what community members expect from the CAP (30).
- Little attention has been paid to assessing the community's sense of the quality of the CAP relationship or what could be done to improve it; (31) and
- Few frameworks take into account how the community negotiates the imbalance of power inherent in the CAP (32).

**Figure 2:**
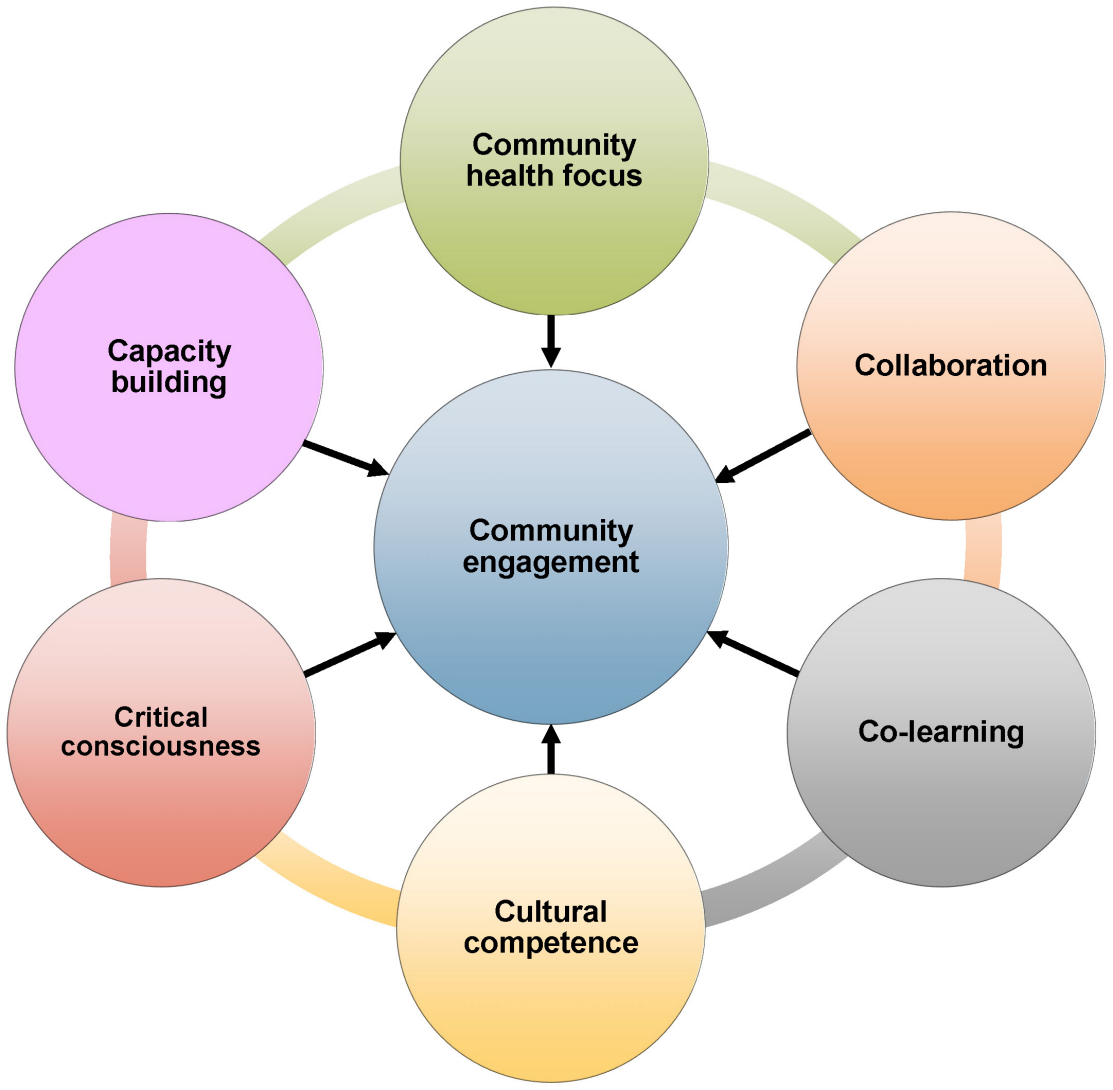
6 C model of community engagement.

To address these deficiencies, the authors first reviewed the existing literature. The databases searched were PubMed, CINAHL, Web of Science, PsycINFO, and Cochrane Central Register of Controlled Trials to identify studies describing models of community engagement, conducted between 2020 and 2025. The search period from 2020 to 2025 was chosen to capture the most relevant and contemporary literature on community engagement. This relatively recent timeframe allowed for a robust analysis of trends, gaps, and developments in the field over the past 5 years. The search identified 12 articles published since 2020 that summarized community engagement models (Table 1). Over 20 models were acknowledged in the search. There was a universal focus on partnership processes and levels of engagement. All these models were essentially “top down” approaches, initiatives created at the institutionalized strategic level (33). As such, these community engagement models fell short of genuinely addressing the perspectives of the community. Conversely, we were particularly interested in characteristics that could be measured by both community members and scientific researchers. We perceived there were three critical characteristics that were either missing or understated in existing published frameworks as well as three others that were needed to move CAPs toward a more shared leadership style. The first three were community health focus, collaboration, and co-learning. We added three others that we had observed in our own work. These were cultural competence, critical consciousness, and capacity building. This model, named the “6C model of community engagement” is depicted in Figure 2.

**Table 1:** Overview of the reviewed resources.

**Authors**	**Country**	**Type of source**	**Summary**
Torró-Pons et al. (2024)	Spain	Systematic review	• Community participation was conceptualized as citizen science. • Citizen science studies in nursing were notably recent (2017–2023). • A total of 13 nursing studies were identified. • Five research areas were identified, with environmental health being the most predominant.
MacFarlane et al. (2020)	Ireland	Research paper	• Social science concept of participatory spaces was used to describe community participation to develop primary care services. • Shared decision-making, community participation and Public and Patient Involvement (PPI) emphasized.
Viglione et al. (2023)	USA	Theoretical paper	• Practice-based research networks (PBRNs) are grounded in community engagement and community-based participatory research (CBPR) practices
Ayton et al.	Australia	Qualitative study of barriers to Consumer and community involvement (CCI)	• Thematic analysis was guided by the Capability, Opportunity and Motivation and Behavior model (COM-B) • Lack of time and resources for CCI, challenges in finding consumers for projects and a perceived lack of evidence of the impact of CCI were barriers.
Maillet et al. (2025)	Canada	Qualitative case study	• Complex adaptive systems approach • To implement and manage an innovation in a healthcare organization, it is fundamental to foster coevolution at operational, tactical and strategic levels, as well as with the external environment.
Heumann et al. (2022)	Germany	Integrative review	• Aim was to develop a conceptual framework for nurses' involvement in community participation processes • Data reveals nurses are involved in facilitating patient and community participation: (1) sharing understanding of health problems and needs, (2) developing resources and facilitating patient education for self-management, (3) raising patients' voices as an advocate in service development and (4) supporting individual and community networks.
Thomson et al. (2024)	UK	Mixed methods analysis of focus group data	• Ten online focus groups were conducted with research teams from across the UK exploring the successes and challenges of partnership working to tackle health inequalities using collaborative approaches to community-based research. • Successes included employing practice-based and arts-based methods, being part of a research project for those not normally involved in research, sharing funding democratically, building on established relationships, and the vital role that local assets play in involving communities.
Sheldon et al. (2024)	UK	Qualitative (thematic analysis)	• CBPR model • Several barriers experienced to healthcare access and utilization, including language accessibility, staff attitudes and awareness, mental health and stigma, continuity of support, and practical factors such as ease of service use and safe spaces.
Shea et al. (2022)	USA	Qualitative focus groups	• Qualitative research (focus groups) was conducted to identify (i) the potential motivators and barriers to study participation across different races and ethnicities; (ii) preferred delivery of education and information to support healthcare decision-making and the role of the community. • Need for pharmaceutical companies and other entities to authentically engage in strategies that build trust within communities to enhance recruitment among diverse populations.
Manalili et al. (2022)	Canada	Focus group discussions with patients/caregivers to obtain their perspectives on their values, preferences and needs regarding patient centered care (PCC).	• Participatory action research approach using “community brokers” • Initiatives were identified to improve PCC, such as codesigning innovative models of training and evaluation of healthcare providers
Khatri et al. (2024)	Australia	Scoping review	• This scoping review synthesized existing evidence on the interlinkage between community health programs (CHPs) and the community health system and beyond for delivering and utilizing primary health care (PHC) services toward universal health coverage (UHC). • A total of 81 studies were included in the final review. Studies described CHPs as foundations for community health system readiness for PHC services.
Shinkai et al. (2025)	USA	Scoping review	• Identify, describe, and discuss models and experiences of citizen science in dentistry and community oral health, with a focus on moderate through large involvement of citizens in the research process. • 16 studies were included for data charting and analysis. All studies were conducted in North America, Europe. • Most studies targeted socially disadvantaged local minority groups (e.g., indigenous people, immigrants, and low-income families) with several health problems (e.g., oral diseases, bad habits, and poor access to oral health care services). • Citizen science in dentistry is still evolving

### Community health focus

This condition addresses the shared process of establishing community health priorities. This is the first step in determining how and where resources should be targeted (13). Identifying community health priorities is often the first task of a CAP and while it seems fairly obvious, it can be a difficult initial step. There are often numerous health issues that need to be addressed in a specific community and there may be differences in community-identified and academician-identified health problems. Likewise, either partner/both partners may verbalize genuine community issues or focus solely on personal priorities. “Insiders” and “outsiders” are likely to prioritize different issues. Ultimately, a coalition of CAP members should meet to select and set priorities. There are a number of potential priority-setting approaches available (34–36). Often several priorities are preliminarily selected. While factors such as the availability of external funding often drive the final selection of priority areas, it is important to include pragmatic issues of concern to community members, for example, crime rates in residential areas, domestic violence or issues with air and water pollution.

### Collaboration

Collaboration is a key element in all stages of community engagement. In CER, academic researchers and community members are equal partners who work together in all phases of the research process. Collaboration is particularly a feature of more egalitarian forms of community engagement, where trust and subsequently bi-directional communication are the norm (26). Despite the emphasis on collaboration in the partnership, little attention is drawn in the literature to a community's sense of the quality of the CAP relationship or what could be done to improve it (37–39). This lack of community perspective indicates that more work is needed to create *authentic* equitable CAPs.

### Co-learning

Unlike the existence of a traditional educational hierarchy between teacher and student, in CER, community members and scientific researchers learn from each other. Both academic and community partners must acknowledge the presence of different “ways of knowing” (40) including empirical (science), esthetic (art), personal (personal knowledge), and ethical (moral knowledge). Community wisdom refers to the collective knowledge, skills, insights, and experiences that residents within a community possess and bring to bear on solving collective challenges. This type of “knowing” is a complement to the specialized scientific knowledge of researchers and encourages both partners to work together to understand, solve problems, and construct their own hybrid form of knowledge. Tossas et al. (41) refer to the dissemination of knowledge in CER as the “community to bench model,” where research is carried out by community members and communicated to health care researchers at the “bench.” Transmission in this manner is theoretically much faster and the findings are more pragmatic.

### Cultural competence

In the context of CER, culture is a multi-faceted concept that may refer to age or generation; gender; sexual orientation; occupation and socioeconomic status; ethnic origin or migrant experience; religious or spiritual beliefs; and/or ability (42). Because communities are often composed of individuals representing a variety of cultures, cultural competence requires the ability to effectively interact, work, and develop meaningful relationships with people of various cultural backgrounds (43).

Beyond just competence, scientific researchers have a responsibility to promote cultural safety (44) among their community partners, preventing any action which diminishes, demeans or dis-empowers the cultural identity and wellbeing of an individual during any component of the research process (45). Investigators need to be aware that community partners may be hesitant to meet in an academic setting, which may require the use of multiple forms of public transportation or the added expense of taxis from their neighborhood. Community health workers/*promotoras* noted that they often experienced “*miradas sucias*” (dirty looks) from the nursing students they encountered near the campus. This observation prompted the initiation of *Buen Vecino* (Good Neighbor), a clinical partnership with the School of Nursing and the Mexican Consulate, to familiarize students with the health needs of Mexican immigrant families.

### Critical consciousness

Paulo Freire, the Brazilian educator and philosopher (46) first coined this term to help explain how individuals can understand and change social, political, and economic structures in society. Critical consciousness theory argues that oppressed groups are disempowered and dehumanized through objectification and silencing. Freire believed that through theory, reflection, and action, it is possible for individuals to work together and to see beyond their own personal experience and understand the diverse experiences of others. Critical consciousness comes about through the concept of praxis, a recursive process of discussion, reflection, and action (46). Whether an individual is a member of a privileged or oppressed group, development of critical consciousness can “help individuals to understand their role in a system of oppression (47).” For this reason, critical consciousness is an essential element of CER.

### Capacity building

Capacity building is defined as the improvement of an individual's or organization's ability to produce or perform (48). As such, capacity building can be seen as the end product of CER, particularly when it involves changes in policy (49). But capacity building can occur in an incremental fashion as well and should be seen as an ongoing process. In CER, capacity building should occur among both community members and scientific researchers (26). Sometimes, capacity building in one partner can appear to take place at the expense of the other. Our early experiences training *promotoras* taught us that some women chose to use the training they received as a steppingstone to other career opportunities. In many instances, these opportunities offered higher pay and more job security than their academic partners could provide.

Although the literature is clear about what scientific researchers want from their community partners, many community engagement theories are less specific about what community members expect from researchers. In general, the literature suggests community members want verification that: (1) the researcher is capable of performing the research; (2) the research is expected to benefit the community or that it is useful to the community in ways that justify its participation; and (3) the community can trust the researcher to pursue the particular research project in a manner respectful of the community (50). The 6C model of community engagement is a step in the right direction to address the needs of community partners.

## Results

Having identified the six critical characteristics of community engagement, the next step is to have community and academic partners evaluate: (1) their perceived location on the Clinical and Translational Science Award Consortium community engagement continuum (Figure 1); as well as (2) the presence and (3) strength of each of the six measures of community engagement in the CAP. To do so, both groups will answer the following questions using a 5- point Likert scale (Table 2).

**Table 2:** Evaluation of community engagement process.

**How often have you seen examples of this characteristic at work in this Community Academic Partnership (CAP)?**					
**Characteristic**	**Never (1)**	**Rarely (2)**	**Sometimes (3)**	**Frequently (4)**	**Always (5)**
Community health focus- Selection of a community health priority area					
Collaboration-Community members and academicians are equal partners					
Co-learning-Community members and academicians learn from each other					
Cultural competence-Community members and academicians demonstrate a respect for various cultures in the community and their beliefs					
Critical consciousness-individuals work together and see beyond their own personal experience to understand oppression					
Capacity building-Both community members and academicians experience an improvement in their lives					
**How would you rate the quality of each of these characteristics of community engagement in this CAP?**					
**Characteristic**	**Poor (1)**	**Fair (2)**	**Good (3)**	**Very good (4)**	**Excellent (5)**
Community health focus- Selection of a community health priority area					
Collaboration-Community members and academicians are equal partners					
Co-learning-Community members and academicians learn from each other					
Cultural competence-Community members and academicians demonstrate a respect for various cultures in the community and their beliefs					
Critical consciousness-individuals work together and see beyond their own personal experience to understand oppression					
Capacity building-Community members and academicians experience an improvement in their lives					

How often have you seen examples of this characteristic at work in this CAP?

- Never
- Rarely
- Sometimes
- Frequently
- Always

How would you rate the quality of each of these characteristics of community engagement in this CAP?

- Poor
- Fair
- Good
- Very good
- Excellent

Total scores for each construct run from 6 to 30; higher scores indicate higher frequency/higher quality of examples of community engagement. Once the results are tabulated, CAPs will then meet to discuss the similarities and differences between community members and scientific researchers in their findings, using Group Concept Mapping (GCM) (51). A community interpretation session will then be held to disseminate the findings. The tools and approaches used in GCM help partners articulate issues, solve problems, and develop results that support measurable progress (52, 53). Results can be used to determine what can be done to improve equality and the balance of power among the partners.

## Discussion

The 6C model of community engagement is a comprehensive attempt to promote community involvement in the research process. The model was developed based on “real world” collaborative impact stores to address health equity challenges in immigrant communities and has potential utility in other marginalized communities that experience health disparities and inequities. Community and scientific partners both participate in a mixed method evaluation of the partnership, creating a dual “top-down bottom-up” approach to engagement. It is expected that CAPs who utilize this process will be more likely to identify what community members expect from researchers, and to take steps to develop egalitarian relationships, characterized by shared leadership and attention to broader health outcomes in the community. As Woodrow Wilson said:

*There must be, not a balance of power, but a community of power; not organized rivalries, but an organized peace*.

Currently, the 6-C model is undergoing formal testing and verification procedures, to determine the psychometric properties of the evaluation measures. These measures can be implemented as part of a CAP activity, in either a formative and/or summative evaluation and then followed up with a GCM activity to compare the similarities and differences between community members and scientific researchers. In preliminary testing, these mixed-methods tools have been found to be simple, pragmatic and potentially very effective in assessing the effectiveness of the CAP.

## Data Availability

The original contributions presented in the study are included in the article/supplementary material, further inquiries can be directed to the corresponding author.
